# Ergotine in Epidemic Diarrhœa

**Published:** 1857-09

**Authors:** M. Massola


*Ergotine in Epidemic Diarrhœa, by M. Massola* (*Gaz. Hebdom de Med. et Chir.*)—In a communication to the Academy of Medicine in Paris, M. Massola states that he found great benefit from the use of ergotine in the fatal epidemic diarrhœa, which prevailed so extensively among the Sardinian troops, in the recent Campaign in the Crimea. From fifteen to twenty grains were added to ℥viii of water, and a table-spoonfull of this mixture was given every half hour. M. Massola states that astringents, tonics, opiates or stimuli, were of little avail as compared with the ergotine.—*Half Yearly Abstract of the Medical Sciences.*

